# HIV care interruptions, mental health, and the potential for mHealth interventions among adolescents and young adults with HIV in Uganda

**DOI:** 10.1186/s12981-026-00853-w

**Published:** 2026-02-03

**Authors:** Julian Adong, Nicholas Musinguzi, Denis Nansera, Henrietta Nayiga, Angella Kankunda, Lisa M. Bebell, Jessica E. Haberer, Elias Kumbakumba

**Affiliations:** 1 Department of Paediatrics, Faculty of Medicine, Mbarara University of Science and Technology, P.O. Box 1410, Mbarara, Uganda; 2 Global Health Collaborative, Mbarara University of Science and Technology, Mbarara, Uganda; 3 Mbarara Regional Referral Hospital, Mbarara, Uganda; 4 Centre for Global Health, Massachusetts General Hospital, Boston, MA USA

**Keywords:** HIV, Adolescents, mHealth, Mental health, COVID-19

## Abstract

**Objective:**

HIV care interruptions contribute to adverse outcomes among adolescents and young adults with HIV (AYWH) and may occur due to structural barriers as well as comorbidities (e.g., mental health issues). This study characterizes a cohort of AYWH, examines the frequency of care interruptions, and assesses mental health issues during and after the COVID-19 pandemic while exploring mobile health (mHealth) potential.

**Methods:**

Using a retrospective and prospective cohort study design, we enrolled AYWH at Mbarara Regional Referral Hospital and assessed missed visits using the timeline follow-back method (24 months). Mental health was evaluated using the Centers for Epidemiological Disease Scale-Depression (CES-D; >15 considered significant) and a locally validated anxiety/psychosocial distress scale (score 0–100) at enrolment, three and six months. Access to mobile phones, smartphones and internet was also assessed.

**Results:**

Of 86 participants (mean age 18.6 years, 51.2% male), 89.5% had a viral load of <400 copies/ml. At enrolment, 53% had depression, with mean anxiety/psychosocial distress of 36.7. AYWH missed 19.0% of clinic visits, 3.2% of ART pickup visits, and 5.1% of laboratory visits, with no clear variation by pandemic phase. Depression and anxiety decreased significantly over 6-months (β=– 0.46; 95% CI – 0.73, – 0.19; p<0.001) and (β=– 1.25; 95% CI – 1.65, – 0.86; p=0.001) respectively. Most AYWH (59%) had mobile phone access, with 67% of those owning a smartphone and 71% having daily internet access.

**Discussion/conclusion:**

AYWH frequently missed clinic appointments, regardless of pandemic phase. Mental health symptoms were initially high, but decreased over time. Most AYWH had access to phones and the internet. Conclusion: To ensure continuity of HIV care and mental health support even during such disruptions, mHealth interventions may offer a viable solution and warrant further research.

## Introduction

Over 4 million adolescents and young adults with HIV (AYWH) live in sub-Saharan Africa (sSA) [1]. Despite the successful rollout of highly active combination antiretroviral therapy (ART) throughout sSA and significant strides in HIV care and treatment outcomes [1], AYWH continue to have significantly higher rates of treatment failure and mortality compared to other age groups living with HIV [2]. Globally since the peak of HIV in 2023, overall AIDS-related mortality had declined by about 64%, but only 10% among AYWH [3].

In general, adolescence represents a vulnerable time due to the typical developmental stages of adolescence having distinct biological, psychological and social differences that affect chronic HIV care [4]. AYWH are even more disadvantaged; in addition to the challenges faced by all adolescents, they live with a chronic stigmatizing disease and deal with the lifelong need for medication and regular medical appointments. Moreover, many have complex social circumstances, including frequent change of caregivers, disclosure of HIV status to peers and family members and a school system that is not tailored to their needs [5–7]. Compared to other age groups living with HIV, AYWH face greater self-regulatory challenges and have limited coping skills; AYWH are thus more prone to the impact of stigma, mental health issues, and socio-behavioral challenges, including substance use, sexual and reproductive health (SRH) concerns, and medication adherence [8]. These difficulties are often complicated by treatment interruptions that limit clinician efforts to improve AYWH outcomes. Treatment interruptions result from AYWH missing appointments, which may be as a result of social reasons such as; having multiple caregivers, lack of household structure, non-disclosure, and economic reasons such as family and personal poverty, thus inability to cover transport and other treatment-related costs; missing appointments may however also result from structural barriers outside the control of AYWH.

In March 2020, additional challenges for AYWH HIV care arose when worldwide concerted efforts to curb the spread of COVID-19 led to inter- and intra-country travel restrictions. In Uganda and elsewhere in sSA, these restrictions included periods of complete cessation of movement, the implementation of stay-at-home orders, and bans on all forms of social gatherings, including hospital visits, except in emergency cases. Widespread physical distancing recommendations and social gathering limitations further reduced AYWH access to health appointments, peer meetings, and school attendance, which are important sources of psychosocial support for AYWH [9, 10]. The increased social isolation, time away from school, and lack of structured routines during this time may have worsened symptoms of depression and anxiety among AYWH that could manifest in the long-term as loss of virologic suppression, further exacerbating morbidity and mortality.

Because of their vulnerable developmental stage, disruptions to routines and health care services for AYWH may exacerbate medical and social issues to a much greater degree in AYWH compared with their adult counterparts [11]. The initial effects of service disruptions and social isolation on AYWH HIV care and mental health were described at the very start of the pandemic; however, post-pandemic effects have not yet been described or quantified in detail [12].

Mobile health (mHealth), defined as the use of wireless technologies to deliver health services, may represent a potential solution to address disruptions in care. However, implementation strategies and outcomes vary across AYWH [13, 14]. Therefore, assessing the availability of mobile devices and internet access in semi-rural Uganda is essential to harness the potential of mHealth and help inform implementation strategies.

In this analysis, we characterize a cohort of AYWH in semi-rural southwestern Uganda and describe the frequency of the interruptions in HIV care and treatment during the COVID-19 pandemic. We also describe how symptoms of depression and anxiety experienced by AYWH evolved during and after the pandemic period and the association of these mental health issues with interruptions in care. We further assess the potential for mHealth to bridge the interruptions in care by evaluating access to mobile phones, smartphones, and the internet.

## Materials and methods

### Study design and setting

We conducted a combined retrospective and prospective cohort study of AYWH at the Mbarara Regional Referral Hospital’s Immune Suppression Syndrome (ISS) clinic. The ISS clinic provides comprehensive HIV care to AYWH who comprise more than half of the 2,500 pediatric HIV clinic patients, totaling about 1700 AYWH. The clinic’s standard of care is guided by the Uganda Ministry of Health guidelines, which are locally contextualized extrapolations of WHO recommendations. Current guidelines recommend the first-line regimen as TDF/3TC/DTG, and over 90% of AYWH in this clinic are on this regimen, with some taking other combinations due to various reasons. Clinical visits occur every 2–3 months for AYWH with good adherence, and more frequently, such as monthly, for those with adherence challenges. Laboratory visits usually occur every 6 months, and ART pick-up visits occur at varying intervals (1–6 months) between clinical visits, depending on the specific circumstances of each AYWH. Screening for mental health issues, SRH issues, and alcohol use is not routinely carried out.

In March of 2020, the Ugandan government instituted a complete societal lockdown through June 2020, followed by inter-district movement restrictions from July 2020 through August 2021. Despite easing restrictions after this date, schools and non-emergency movement remained under lockdown until January 2022.

We enrolled AYWH between March and July 2022 and followed them for 6 months with visits occurring every 3 months. AYWH were eligible for the study if they were aged 13–24 years, were prescribed a tenofovir diphosphate (TDF)-containing ART regimen (to enable objective adherence monitoring), and spoke English or Runyankole (the local language).

### Sample size calculation and sampling

We powered the study for the primary aim that is not reported in this paper. The primary aim was to detect a 20% difference between baseline and follow-up ART adherence, as measured by tenofovir diphosphate in dry blood spot samples collected from this cohort, with 80% power to detect point estimates. The resulting sample size was initially *N* = 71. We, however, recruited an additional 20% to account for potential attrition and based on published adherence analyses, yielding a final sample of *N* = 86 participants.

Participants were enrolled from a convenience sample with consecutive enrolment until the sample size was achieved. Phone access was not used as an enrolment criterion at baseline.

### Data collection

Research assistants administered structured questionnaires on paper and entered results into a Research Electronic Data Capture (REDCap) database [15]. Data gathered included sociodemographic information (e.g., age, sex, education/literacy) and mobile phone use; medical history was abstracted from ISS clinic charts.

#### Alcohol use

We assessed alcohol use using the Alcohol Use Disorders Identification Test – Consumption (AUDIT-C), modified to cover the prior 3 months. The AUDIT-C is a brief alcohol consumption-screening tool. It consists of 3 questions scored 0–4; a score > 3 for females and > 4 for males is suggestive of problematic drinking [16]. The AUDIT-C has been used in similar settings in Uganda [17, 18].

#### Sexual and reproductive health (SRH)

We developed and administered a structured questionnaire to collect data on unintended pregnancies, coercive sex, condom use and sexually transmitted infections. We used domains recommended by the World Health Organization (WHO) to develop these questions [19].

#### Child abuse measures

We used the International Society for the Prevention of Child Abuse and Neglect (IPSCAN) child abuse-screening tool to evaluate for child abuse [20] The IPSCAN tool has been used in a Ugandan rural population [21].

This tool was interpreted on a continuous scale; higher IPSCAN scores indicate greater experience of physical, emotional, and sexual abuse.

#### ART adherence

We collected ART adherence at enrolment and at six months post-enrolment by measuring:

(1) 30-day recall by self-reported number of missed doses, frequency of adherence as prescribed, and percent of missed doses [22]; (2) clinician recorded ART adherence by chart review, which is measured by estimating the number of missed pill doses, expressed as a percentage.

### Interruptions in care

Using the timeline follow-back (TLFB) method [23], we asked AYWH about the care service interruptions they experienced during the COVID-19 restriction period. The TLFB interview covered the time period between March 2020 and study enrollment (March 2022 to July 2022; approximately 24 months for most participants). The TLFB utilizes calendar recall and recollection of events around important dates and has been widely used to increase accuracy of a variety of self-reported behaviors and events [23]. AYWH were asked to recall scheduled care services including physical clinical visits (clinical visits), visits to pick up ART only (ART pickup visits) and visits to the clinic to have scheduled laboratory tests performed (laboratory visits) during this time period, and whether each visit was completed or missed. For each visit interruption, we asked the participant what they perceived as the cause of the interruption. To account for potential recall and social desirability bias, we corroborated participant self-report by performing chart reviews of ISS clinic records, including physical patient treatment charts/cards; these are cards that capture each visit and subsequent appointment date for a maximum of up to 2 years of follow-up, each is placed in a patient file with a unique clinic ID, and the data captured on this card is entered into an electronic data base which we also accessed to corroborate self-report. we also reviewed pharmacy refill data (both electronic and paper records). Data abstracted included clinical visits scheduled and marked as completed in the participant chart review and/or participant report, scheduled laboratory visits (chart review), and ART pickup visits (chart review and pharmacy refills). We noted whether these scheduled visits were marked as completed or missed. In cases of discrepancy, we relied on the chart review.

#### Depression

We administered the Centers for Epidemiologic Studies-Depression (CES-D) Scale, a 20-item symptom checklist with each item scored from 0 to 3; a score of > 15 represents significant symptoms of depression. The CES-D has been validated among AYWH and was previously used among AYWH in Uganda [24].

#### Anxiety/psychosocial distress

We used the 25-item anxiety/psychological distress instrument, locally developed and validated in Uganda for use among AYWH [25]. The instrument contains six subscales: anhedonia, depressive anxiety, isolation, suicidal ideation, sleep problems, and somatization. It is interpreted on a continuous scale (scores 0-100); higher scores indicate greater symptoms of anxiety.

#### Statistical methods

We described categorical variables using frequencies and proportions as well as means and standard deviations (SD) for continuous variables. We graphically represented the proportion of missed clinical visits by plotting bar graphs and superimposing a line graph of the variations in pandemic restrictions. We then graphically analysed any variations in missed clinical visits by change in restrictions. Three time periods were identified based on differing levels of pandemic restrictions in place: “Heavy restrictions” was defined as March 2020 through October 2020; “Mild restrictions” was defined as November 2020 through December 2021; and “No restrictions” was defined as January 2022, through March 2023, when all participant follow-up ended. We calculated the proportion of missed visits per participant per study visit. Using the Wilcoxon-ranksum test, we tested for the difference in mean percentage missed visits among participants with and without clinically significant symptoms of depression and anxiety.

To assess the progression of depression and anxiety through the follow-up period, we fit linear generalised estimating equation models with depression and anxiety as the outcome and time (in months) as our predictor variable. Additionally, we assessed for non-linearity by including the quadratic term and rejected linearity if the p-value for the quadratic term was significant.

### Ethical clearance

The study was approved by the institutional ethics review boards at Mbarara University of Science and Technology (MUST-2021-66), Mass General Brigham IRB (2021P002231/MGH) and the Uganda National Council for Science and Technology (HS1686ES). All participants aged 18 years or older provided written informed consent prior to enrollment. Participants aged < 18 years provided assent, and parental consent was obtained.

## Results

### Participant characteristics

A total of 86 participants were enrolled, of these 44 (51.2%) were male, the mean age was 18.6 (standard deviation [SD] 3.0) years, 80 (93.0%) were literate in either English or Runyankole, and the majority (70%) identified as students with half (50%) having completed secondary school education (Table 1). The average duration on ART at enrollment was 12 (SD 4.1) years, and 77 (89.5%) had HIV viral load counts of ≤ 400 copies/ml (Table 1). Over a third (35%) of AYWH reported a sexual encounter in the prior 24 months. Of these, 18/30 (60%) used a condom at each sexual encounter and 8/30 (27%) reported more than one sexual partner. Of the AYWH who reported having sex, 4/30 (5%) reported coercive sex, and 7/30 (23%) self-reported having a sexually transmitted infection (STI). One out of every two (50%) AYWH had experienced at least one form of child abuse; 83% had experienced violence, 89% victimization, 72% neglect, 61% physical punishment, and 50% sexual abuse using the ICAST-C tool (Table 1).

**Table 1:** Baseline demographic and clinical characteristics of AYWH enrolled between March and June 2022 (*N* = 86)

Variable	*N* (%)
Sex	
Male	44 (51.2)
Age Mean [SD]	18.6 (3.0)
Literate in English or Runyankole	80 (93.0)
Average ART duration, years Mean (SD)	12 (4.1)
Chart recorded adherence ≥ 95%	79 (92.9)
Self-reported number of days ART missed in the last 30 days	
None	55 (64.0)
One or more days	31 (36.0)
Self-reported perceived ART adherence	
Excellent	19 (22.1)
Very good	41 (41.7)
Good	21 (21.4)
Fair and/or poor	5 (5.9)
Self-reported frequency of taking ART	
Always	40 (47.1)
Almost always	31 (36.5)
Usually	8 (9.4)
Sometimes and /or rarely	6 (6.1)
Viral load ≤ 400 copies/ml	77(89.5)
Mental health, SRH and child abuse	
Symptoms of depression (CES-D), Mean [SD]	17 (7.4)
Symptoms of depression > 15	
No	40 (47.1)
Yes	45 (52.9)
Psychosocial distress/anxiety, Mean [SD]	36.7 (8.9)
Had sex since the pandemic started	30 (34.9)
More than one sexual partner*	8 (27)
Always use condom at last sexual encounter*	18 (60)
Had sex for money*	4 (4.7)
Coercive sex*	4 (4.7)
Self-reported STI*	7 (23)
ICAST-C subscales	
Violence	71(83)
Victimization	77(89)
Neglect	62(72)
Physical punishment	53(61)
Sexual abuse	43(50)

*= n = 30

### Mobile phone and internet use

Overall, nearly two-thirds (51/86, 59%) had mobile phone access, of whom 34/51 (67%) had smartphone access and 36/51 (71%) had daily mobile internet access, 32/51 (63%) reported receiving messages and 33/51 (65%) reported sending messages. (Table 2). Mobile phone access was not associated with participant sex or literacy, but was associated with older age i.e. age 18–24 years (P = < 0.001). Older AYWH reported significantly greater access to cell phones (48/51/86 [91%] of AYWH aged 18–24) than younger AYWH (3/51 [6%] of AYWH aged 13–17, P = < 0.001).

**Table 2:** Characteristics of baseline mobile phone use and internet access among AYWH who had access to a mobile

Phone (*n* = 51)	
Shared mobilephone	10 (19.6)
Have access to smartphone	34 (66.7
Phone is turned on most of the day	41 (80.3)
Have access to mobile internet	36 (70.0)
Receive several messages/day	32 (39.0)
Send several messages/day	33 (64.7)

### Missed scheduled visits

**Fig. 1:**
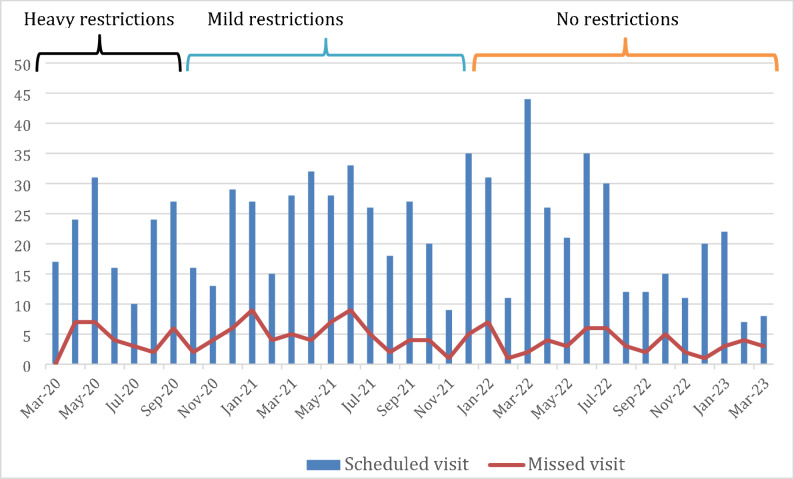
Graph showing scheduled and missed clinical visits from March 2020 through March 2023. Ugandan total societal lockdown occurred between March 2020 and July 2020 (heavy restrictions), partial lifting of restrictions between August 2020 and December 2021 (mild restrictions), and, subsequently, the resumption of normalcy in January 2022(no restrictions)

Of a total of 811 clinical visits (both by chart review and/or participant report) scheduled for all participants between March 2020 and the date of the 6-month follow-up study visit interview, 152 (19.0%) were missed (Fig. 1). Of 811 total ART pickup visits scheduled, 26 (3.2%) were missed, while of 453 scheduled laboratory visits, 23 (5.1%) were missed. From a graphical analysis, the missed clinical visits had no clear association with the evolution of the pandemic restrictions (Fig. 1). Of all the missed visits, only missed laboratory visits were significantly associated with a viral load > 400 copies/ml (*P* = 0.02).

### Mental health and socio-behavioral factors

**Fig. 2:**
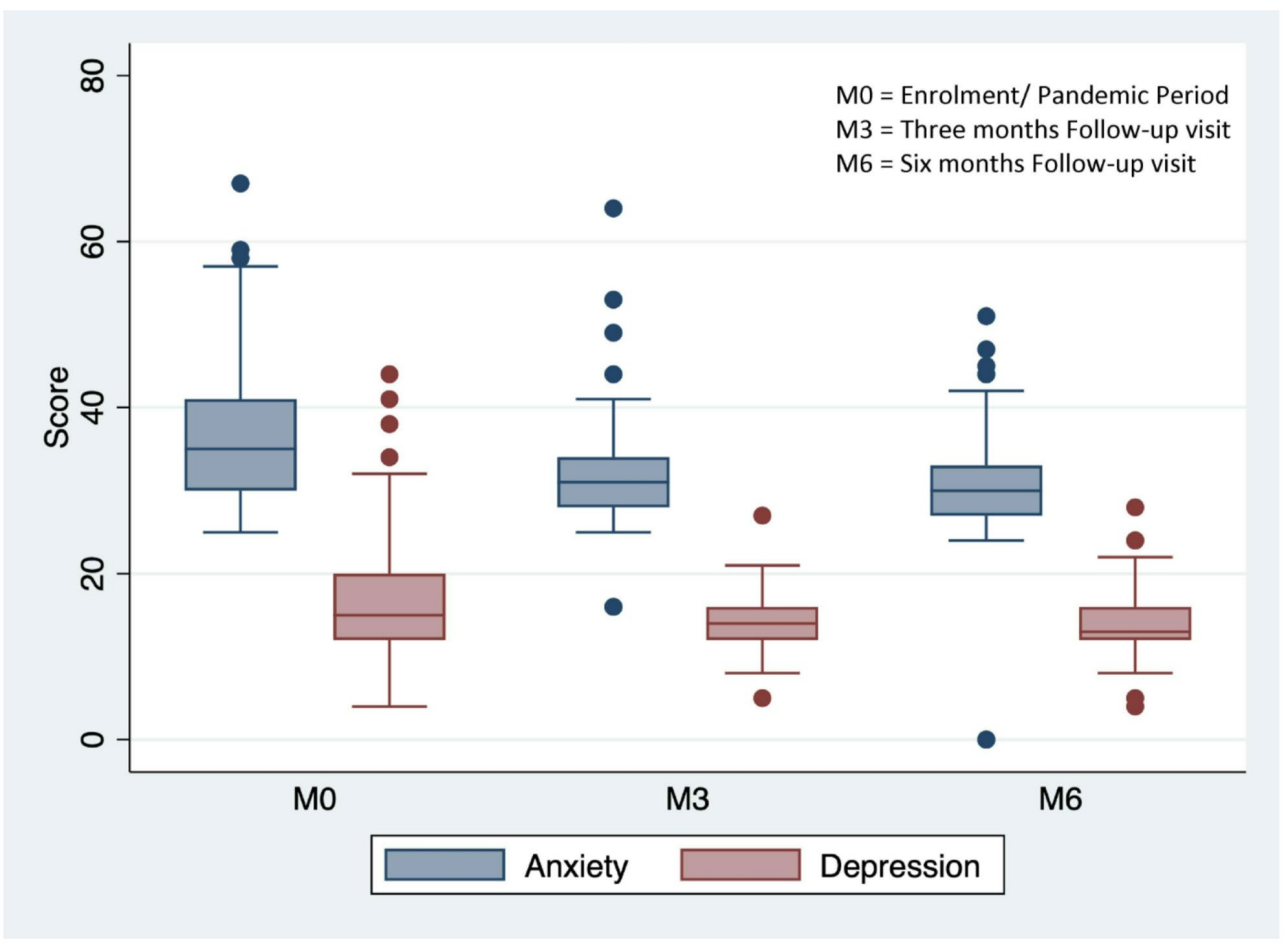
Box plot showing decreasing symptoms of depression and anxiety/psychosocial distress from enrolment to three and six months

At enrolment, the prevalence of clinically significant symptoms of depression was 45/85 (53%, Table 1). Symptoms of depression were similar by biological sex (50% of females vs. 49% of males); The mean anxiety/psychosocial distress score was 36.7 (SD, 8.9, Table 1). Both CES-D (symptoms of depression) and anxiety/psychosocial distress scores significantly decreased over the 6-month prospective follow-up period (Fig. 2): β=– 0.46; 95% CI – 0.73, – 0.19; *p* < 0.001 for depression and β=-1.25; 95% CI – 1.65, – 0.86; *p* = 0.001 for anxiety). The proportion of all missed visits (clinical visits, ART pickups, and laboratory visits) did not vary by clinically significant symptoms of depression or anxiety symptoms.

## Discussion

We found high rates of missed clinical, but not laboratory or ART pickup, visits among AYWH during the COVID-19 pandemic, with no clear variation related to pandemic restrictions. AYWH had very high rates of depression and anxiety symptoms, which decreased throughout the six-month follow-up period but were not significantly associated with missed clinical visits.

Similar to our study results, disruptions in care were reported by other HIV clinics in sSA, with higher rates of missed physical clinical visits, but minimal impact on ART pick-ups during the COVID-19 pandemic. For instance, a case report from a major HIV clinic in eastern Uganda revealed that approximately 25% of all visits (clinical visits, ART pick-up visits and laboratory visits) were missed, with higher missed clinical visits [26] compared to the observed rates in our study. Notably in this study, younger individuals aged 20–24 were more prone to missing clinical visits. There were low instances of missed ART pick-ups during the pandemic, as seen in our results and other reports. This finding could be attributed to the adaptations made in healthcare delivery models across HIV care facilities in response to the pandemic. During the COVID-19 pandemic, many facilities adjusted their services by extending the duration of each ART refill, implementing community-based approaches to ART distribution, leveraging existing differentiated care models, utilizing mobile phones for coordinating remote ART provision, and reducing the frequency of scheduled ART pick-up and laboratory visits [27]. These adjustments likely contributed to the reduced number of missed laboratory appointments and ART pick-ups. It is worth mentioning that this case study did not assess participant levels of depression or anxiety.

The high levels of depression and anxiety we identified were somewhat unexpected. Studies conducted before the pandemic indicate that the prevalence of depression among AYWH ranged from 25 to 37%, lower than what we observed in our study [28, 29]. Early narrative analyses of studies conducted at the onset of the pandemic reveal an increase in social isolation among AYWH, which was linked to psychological distress in the form of anxiety and depression [30, 31]. Our observed higher level of rise in distress could be attributed to the absence of psychosocial support usually provided through face-to-face peer interactions, counseling sessions, and healthcare worker engagements during in-person clinical visits. Additionally, the loss of support from attending school and the structured nature of daily life might have contributed to the observed surge in anxiety and depression symptoms [32] .

It is noteworthy that we did not find a correlation between symptoms of depression and anxiety and missed clinical visits. This lack of association could be because these symptoms may be triggered by factors such as reduced social interactions and school closures, rather than solely by disruptions in HIV clinical care [27]. Interestingly, as AYWH gradually resumed their “normal life” routines, including returning to school and accessing in-person services, we observed a decline in symptoms of depression and anxiety during the follow-up period.

In our study sample of AYWH, we observed high rates of mobile phone and internet access, consistent with findings from other studies conducted among youth with HIV in Uganda. The Rakai cohort, one of the longest-running observation cohorts in Uganda that assesses participant demographics every 12–18 months, found a prevalence of phone ownership of about 58% among 10,618 participants aged 15–19 years, a figure almost identical to ours [33, 34]. Both the Rakai cohort and our study population are similar in that they are peri-urban study sites; however, a small study in an urban setting also found a similar proportion of phone access [35]. The application of mHealth, which involves using mobile wireless technology for healthcare purposes, has been demonstrated to be both acceptable and feasible among AYWH in various research studies [36]. However, there have been conflicting findings regarding its efficacy on clinical outcomes, underscoring the necessity for further investigations to evaluate its effectiveness in larger cohorts of AYWH. As noted above, amidst the COVID-19 pandemic, numerous healthcare facilities modified their HIV service delivery strategies to include remote ART distribution facilitated through mobile phones [27]. This adaptation highlights the critical importance of considering remote service provision as a complementary approach to traditional in-person services whenever feasible.

Our data highlights the high occurrence of sexual and reproductive health (SRH) issues and child abuse that often surface when the protective environment and stability offered by consistent school attendance and daily routines/structure are lacking. The instances of SRH issues and child abuse identified could stem from circumstances of isolation, like being restricted at home with an abusive family member. These conditions can exacerbate the complexities of child abuse and challenges related to sexual and reproductive health encountered by AYWH, potentially resulting in incidents of verbal, physical, and sexual abuse physical, and sexual abuse [21].

Our study was not without limitations. One key limitation is the retrospective nature of the COVID-19 pandemic data we collected, which may have been susceptible to recall and social desirability biases as well as the inability to determine causality. However, we mitigated this risk to some extent by utilizing the TLFB method of recall, which incorporates calendar-based recall to assess events dating back up to 2 years. Additionally, our study had a relatively small sample size, which could have restricted our ability to draw definitive conclusions regarding the relationships between anxiety, depression symptoms, and missed clinical visits. Our study also had a number of strengths: The incorporation of both retrospective and prospective data collection methods enabled us to capture experiences during and after the pandemic, particularly concerning mental health issues; The use of data triangulation techniques also helped to increase accuracy; we cross-referenced visit/appointment data from multiple sources and validated self-reported information through chart reviews.

In conclusion, AYWH missed approximately one-fifth of their clinic visits, regardless of the phase of pandemic restrictions. Symptoms of depression and anxiety were prevalent at the beginning of the pandemic but decreased as restrictions eased. Most AYWH in the study had access to mobile phones and the internet. Despite the fact that most pandemic-related restrictions have been lifted and school and other activities have resumed, the effects of the disruptions in HIV care and support for AYWH are persistent in sub-Saharan Africa. Furthermore, new disruptions may arise due to recent epidemics and social/civil unrest [37, 38]. These findings have significant implications for managing future pandemics and other disruptions. To ensure continuity of HIV care and mental health support even during such disruptions, mHealth interventions may offer a viable solution and warrant further research.

## Data Availability

The data associated with this analysis are available and can be shared if/when needed.
